# Magnetic Quincke rollers with tunable single-particle dynamics and collective states

**DOI:** 10.1126/sciadv.adh2522

**Published:** 2023-06-30

**Authors:** Ricardo Reyes Garza, Nikos Kyriakopoulos, Zoran M. Cenev, Carlo Rigoni, Jaakko V. I. Timonen

**Affiliations:** Department of Applied Physics, Aalto University School of Science, P.O. Box 15100, Espoo FI-02150, Finland.

## Abstract

Electrohydrodynamically driven active particles based on Quincke rotation have quickly become an important model system for emergent collective behavior in nonequilibrium colloidal systems. Like most active particles, Quincke rollers are intrinsically nonmagnetic, preventing the use of magnetic fields to control their complex dynamics on the fly. Here, we report on magnetic Quincke rollers based on silica particles doped with superparamagnetic iron oxide nanoparticles. We show that their magnetic nature enables the application of both externally controllable forces and torques at high spatial and temporal precision, leading to several versatile control mechanisms for their single-particle dynamics and collective states. These include tunable interparticle interactions, potential energy landscapes, and advanced programmable and teleoperated behaviors, allowing us to discover and probe active chaining, anisotropic active sedimentation-diffusion equilibria, and collective states in various geometries and dimensionalities.

## Teaser

Magnetic forces and torques can guide the complex dynamics of active particles driven by electrohydrodynamic rotation.

## INTRODUCTION

Active matter systems consist of a large number of individual agents that absorb energy from their environment and convert it to mechanical forces and motion (*1*, *2*). In recent years, increasing attention has been given to artificial active systems, such as Janus particles (*3*), vibrated polar disks (*4*), and Quincke rollers (*5*–*7*). Quincke rollers have emerged as an important system due to their rich collective dynamics and multitude of emergent states, observed both with solid nondeformable Quincke rollers (*8*–*12*) and rollers made of deformable liquid droplets (*13*, *14*). These emergent states include polar liquids (*8*), vortices (*9*, *10*), Lévy flights in pulsed fields (*11*), and active emulsions of liquid rollers (*14*). Often, the dynamics of these states are fast and can be controlled in situ only by using the same field inducing the Quincke rotation, i.e., the electric field.

On the other hand, magnetic forces and torques have been successfully applied to control soft materials, ranging from individual macromolecules (*15*–*17*) to solid particles (*18*) and even bulk liquids (*19*, *20*). They are also being increasingly used in the context of active matter to energize systems of solid ferromagnetic particles (*21*, *22*) and Janus particles (*23*, *24*) via, e.g., oscillating magnetic fields (*25*–*29*). In addition to rendering passive particles active, magnetic fields have been, to limited extent, used to control the dynamics of activate particles by, e.g., steering them (*26*, *30*).

## RESULTS

**Fig. 1.**
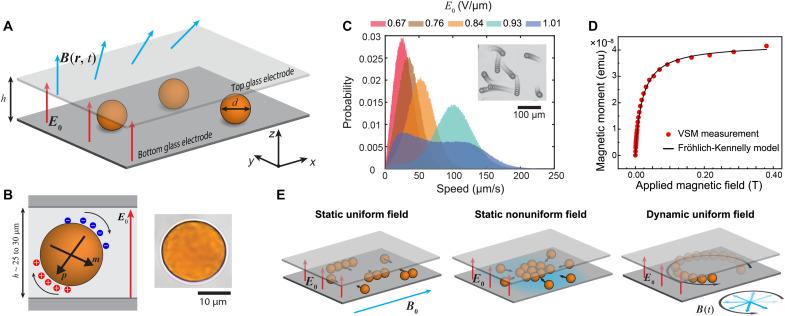
Concept and realization of magnetically controllable Quincke rollers. (**A**) A scheme of colloidal Quincke rollers confined in a horizontal liquid cell in uniform external electric field *E*_0_ and spatially and temporally tunable magnetic field ***B***(***r***,*t*). (**B**) A scheme of a single Quincke roller with the electric dipole ***p*** and magnetic dipole ***m*** indicated, and a high magnification color image of a typical particle (brown color originating from the iron oxide). (**C**) Histograms for translational speeds of the rollers at different driving field strengths (cell height *h* = 29.7 ± 0.9 μm). Inset shows a composite image of the motion of multiple rollers (movies S1 and S2). (**D**) A plot showing the average magnetic moment of a single roller as a function of applied magnetic field strength (red dots) and the best fit of the Fröhlich-Kennelly model (black line). (**E**) A scheme showing different approaches to interacting with the magnetic Quincke rollers.

Here, we report on the development of broadly tunable Quincke rollers based on utilization of magnetic forces (*31*) and torques (*32*). Our system consists of spherical SiO_2_ particles of diameter *d* ≈ 21.6 μm, doped with superparamagnetic iron oxide nanoparticles, that are immersed in a slightly conductive liquid medium (σ ≈ 10^−8^ S/m) (*14*) consisting of n-dodecane with 150 mM of sodium bis (2-ethylhexyl) sulfosuccinate (AOT). The dispersion is incubated in a low-humidity chamber (relative humidity ≈ 5%) to reduce particle charging. Last, it is confined in a quasi–two-dimensional (2D) geometry (Hele-Shaw cell) with two transparent parallel plate electrodes (Fig. 1A).

The particles respond to external electric and magnetic fields by developing electric and magnetic dipoles (Fig. 1B). As with regular nonmagnetic Quincke rollers (*5*, *7*–*9*, *13*, *14*), the electric dipole becomes unstable, and the particles start to Quincke rotate when the applied electric field strength *E*_0_ exceeds the threshold field *E*_Q _≈ 0.6 V/μm (Fig. 1C and movie S1). For *E*_0_/*E*_Q_ between 1 and 1.7, the particles’ translational speed increases with *E*_0_ (Fig. 1C). Above *E*_0_/*E*_Q _≈ 1.7, the particles enter the recently observed “twitching” state (*33*), where the particles display a fast back-and-forth translational motion (movie S2). Magnetically, the rollers display near ideal superparamagnetic behavior with the magnetic moment well approximated by the Fröhlich-Kennelly model $m\approx m_{s}\frac{B}{B+m_{s}/k}$, where *B* is the applied magnetic field strength, *m*_s_ = 4.5 ± 0.2 · 10^−11^ Am^2^ is the saturation magnetization of a single particle, and *k* = 0.24 ± 0.1 · 10^−11^ Am^2^/mT is the slope near the origin (Fig. 1D and fig. S1). The combination of electrically driven and tunable rolling (Fig. 1C) and magnetic responsiveness (Fig. 1D) allows several versatile control mechanisms for the rollers using uniform, nonuniform, and time-varying magnetic fields (Fig. 1E).

**Fig. 2.**
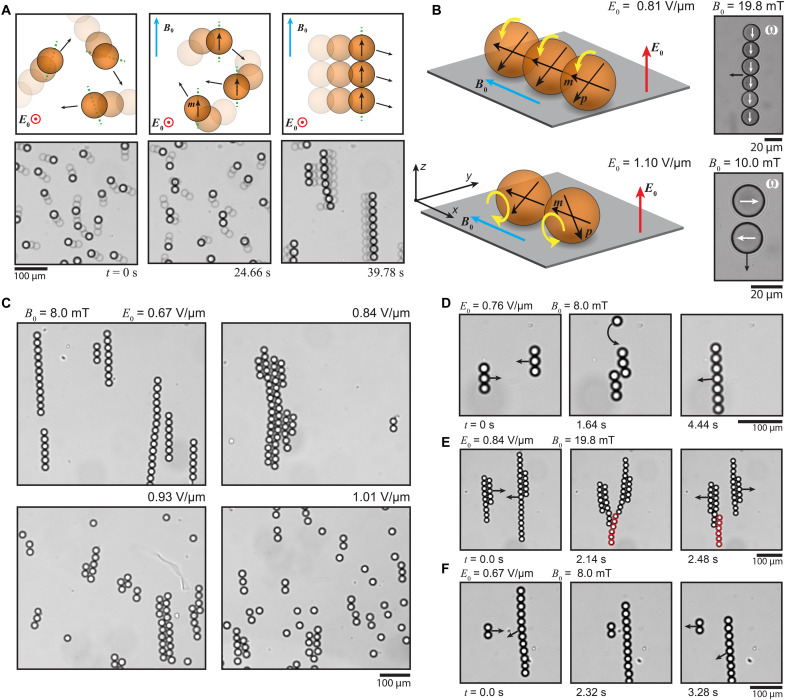
Active chaining of magnetic Quincke rollers in static uniform magnetic fields. (**A**) A scheme (top row) showing the predominant behavior of the active rollers when a uniform in-plane magnetic field is applied and snapshots (bottom row) from the corresponding experimental demonstration (movie S4). The particles’ easy axes are indicated as dashed green lines. (**B**) A scheme of a regular active chain and a snapshot of the experimental realization (top row; movie S5), and a scheme of an anomalous active dimer and a snapshot of the experimental realization (bottom row; movies S6 and S7). The white arrows indicate the axis of rotation. (**C**) Snapshots of the dynamic equilibria between individual rollers and active chains in four different electric fields and constant magnetic field. (**D** to **F**) Time series of microscopy images showing chain-level interactions, including (D) chain fusion, (E) fragment transfer, and (F) scattering (movie S8).

When subjected to a uniform in-plane magnetic field within the Hele-Shaw cell (Fig. 2), the rollers acquire a magnetic moment and experience a torque originating from two sources: dipolar interactions with the neighboring rollers and a weak magnetic anisotropy within the particles themselves (movie S3) that is known to be present in superparamagnetic colloids (*34*). The net torque drives the particles toward aligning their easy axes along the external magnetic field, fixing the axis of the Quincke rotation at the same time. This led to alignment of the rollers, followed by the attractive dipolar forces pulling the rollers together into active rolling chains (Fig. 2A and movie S4). The rolling chains resemble the recently discovered rolling liquid filaments (*14*) but now held together with magnetic forces instead of interfacial tension. When magnetic field is turned off, the magnetic dipolar forces vanish, and the chains fall apart back into individual rollers (movie S4), highlighting the importance of the magnetic forces and the reversibility of the system.

High-speed imaging was used to confirm that the rotation axes of the particles were parallel to the chain direction and, hence, the magnetic field (Fig. 2B and movie S5), as expected from the analogous behavior of individual particles (movie S4) arising from the magnetic anisotropy (*34*), the effect of which is now further amplified by the additional dipolar fields from the other particles within the chain. In addition, the alignment of the axes of rotation minimizes the hydrodynamic dissipation in the chains (*35*).

The rotational frequencies of the chains ranged from *f *≈ 4 to 140 Hz for (fig. S2), as extracted by directly following motion of imperfections on the rotating particles (fig. S3). Because translational velocity *v* ≪ π*fd*, there is notable hovering of the particles between the electrodes (Fig. 1B). The electric repulsion between the rollers originating from the tilted electric dipoles associated with Quincke rotation (*5*, *6*) is overcome by the magnetic attraction to stabilize the chain (Fig. 2B). In rare cases, we observed also anomalous dimer states where the particles’ rotation axes were aligned antiparallel with each other and perpendicular to the magnetic field (Fig. 2B and movie S6). The emergence of a small number of such anomalous dimers suggests that the particles may not be magnetically monodisperse and can have more complicated anisotropies such as easy planes that can support the anomalous dimer state. The dimers tended to translate parallel to the magnetic field, likely due to slight imbalance between the two opposing rollers (movie S7).

The fraction of rollers participating in active chains can be adjusted by tuning the balance between magnetic and electrohydrodynamic forces. The system can be continuously tuned from free rollers only to complete chaining (Fig. 2C and movie S4). Increasing the electric field strength pushes the dynamic equilibrium toward individual rollers due to increasing strength and tilt of the repulsive electric dipoles and increasing hydrodynamic repulsion due to faster rotation. The active chains also exhibit emergent chain-level interactions (Fig. 2, D to F). For example, collisions between chains were observed to lead to full merging of the chains (Fig. 2D and movie S8) or a transfer of a fragment of one chain to another (Fig. 2E and movie S8). Scattering of short chains from larger ones is also possible (Fig. 2F and movie S8).

**Fig. 3.**
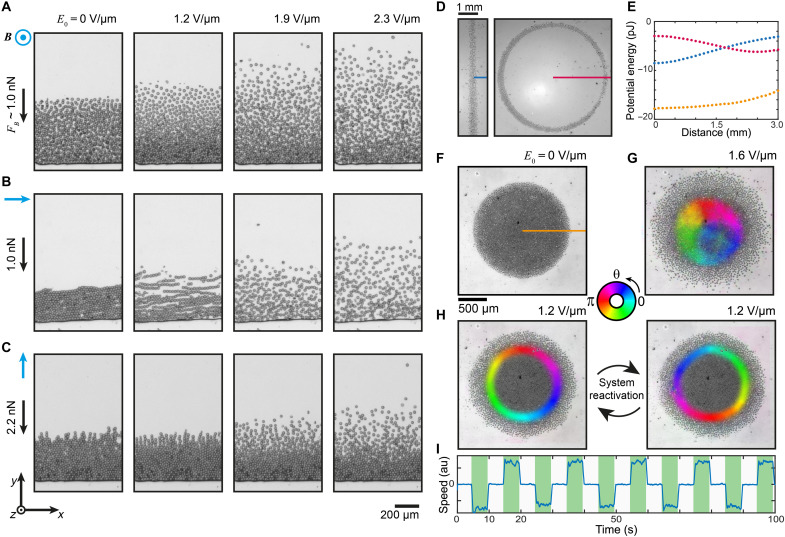
Anisotropic distributions and collective states of magnetic Quincke rollers exploring magnetic potential energy landscapes. (**A** to **C**) Steady-state snapshots of the rollers confined in unidirectional magnetic fields pointing along the (A) *z* axis (movie S9), (B) *x* axis (movie S10), and (C) *y* axis (movie S11) with the field strength decreasing with increasing *y* coordinate. (**D**) Snapshots of rollers confined in quasi-1D linear and quasi-1D continuous (circular) potential energy traps (movie S15), and (**E**) the corresponding axisymmetric magnetic energy landscape profiles. (**F**) A snapshot of an axisymmetric roller condensate in absence of electric field and (**G**) the same with electric field on and a coarse-grained mean velocity field overlaid on the experimental image (movie S16). The color indicates the mean velocity direction while the color intensity correlates with mean velocity magnitude (zero mean velocity is transparent). (**H**) Two snapshots showing the two different orientations of motion. (**I**) Mean speed along a radial line in the condensate as a function of time when the electric driving was periodically turned on and off (movie S17).

When subjected to static but inhomogeneous magnetic fields (Fig. 3), the rollers start to explore the corresponding potential energy landscape *U_B_*(***r***) = −***m***(***r***) · ***B***(***r***) that induces an additional force on the rollers: ***F****_B_* = −∇*U_B_*(***r***). Torques and dipolar forces discussed earlier (Fig. 2) are also present. These magnetic potential energy landscapes and the corresponding force fields are widely tunable: We realized the most elementary case, a constant force, by using a large permanent magnet creating a field decreasing from 190 mT at the one end of the Hele-Shaw cell to 160 mT at a distance of 1200 μm, corresponding to a constant field gradient of 25 T/m (Fig. 3, A and B). In such field, the particles are magnetized to ca. 90 to 95% of saturation *m* ≈ 4.2 · 10^−11^ Am^2^ (Fig. 1B), leading to a constant force of *F_B _*≈ 1.0 nN. This force induced drift to the rollers toward the high-field region, leading to the formation of steady-state particle gradients analogous to sedimentation-diffusion equilibria formed in gravity that have been previously studied for Janus particles (*36*–*38*).

In contrast to sedimentation-diffusion using gravity, the magnetic potential energy landscapes can lead to either isotropic states (Fig. 3A and movie S9) or anisotropic states consisting of active chains (Fig. 3B and movie S10) depending on the direction of the magnetic field. The active chains, rolling along the chain axis, appear to be almost stationary as the active translational motion perpendicular to the chain axis is countered by the magnetic force in the opposite direction, leading to formation of well-defined dissipative anisotropic steady states (movie S10). The minute translational motion along the chain axis originates from the force generated on the magnetic particles by weak but nonzero remaining lateral gradients in the magnetic field. Under stronger field parallel to the field gradient (varying from 435 to 375 mT over a distance of 1200 μm, corresponding to 50 T/m and, hence, *F_B _*≈ 2.2 nN), pairing of standing sedimented active chains was observed (Fig. 3C and movie S11). Similar experiments performed using a smaller concentration of rollers in a two orders of magnitude weaker confining magnetic fields show otherwise analogous behavior but allow better visualization of the single-particle and single-chain dynamics (movies S12 to S14). If the strength of the confining field and field gradient is reduced, then the rollers and active chains distribute more freely in the cell (fig. S4).

More complex potential energy landscapes such as a linear trench (quasi-1D) or a circular racetrack (quasi-1D with periodic boundary) can be created using slab- and ring-shaped magnets as field sources (Fig. 3, D and E, movie S15). A particularly interesting landscape is the quadratic confinement ***F*** = −*k****r*** that can be induced by using an axisymmetric magnet. We observed a highly dense population of rollers in the quadratic confinement that self-assembled into a vortex state (Fig. 3, F and G, and movie S16) without guiding solid walls as used previously (*9*, *10*). In contrast to the previous studies using solid walls, where the roller density is highest near the walls of the confinement, in the quadratic confinement, the density is highest near the center of the confining potential and decreases when moving radially outward (Fig. 3G). Despite of this reversal of roller density as a function of radial position, the vortex state in the quadratic confinement experiences systematic reversal of direction of rotation (Fig. 3, H and I, and movie S17) when the driving is switch on and off periodically (*11*), similar to what has been recently observed using solid confining walls (*10*).

**Fig. 4.**
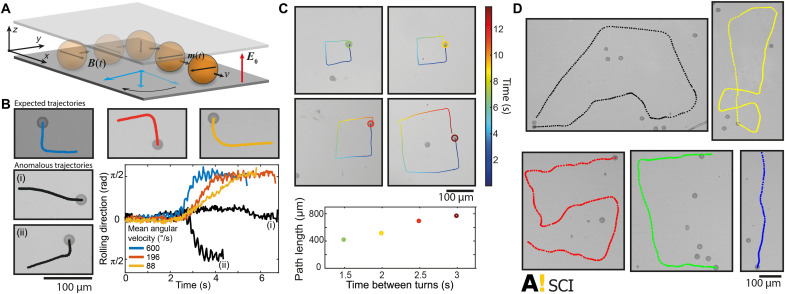
Dynamic control of the rolling direction, programmable trajectories, and teleoperation of the rollers using time-varying uniform magnetic fields. (**A**) A scheme showing a rotation of the rolling direction when magnetic field is rotated. (**B**) Control of rolling direction: Microscopy images showing individual tracked rollers experiencing a 90° clockwise rotation of the external field, leading to three expected 90° clockwise rotations in the rolling directions (colored trajectories) and two anomalous responses (black trajectories). The plot shows the corresponding changes in the rolling direction as a function time. (**C**) Programmable trajectories: Four microscopy images showing individual tracked rollers programmed to perform square trajectories of various edge lengths. The plot shows the full square trajectory length as a function of wait time between the turns. (**D**) Teleoperation: Microscopy images showing tracked rollers teleoperated in real time using an input device to follow complex trajectories, in this case, the Aalto University logo (movie S18).

Last, the magnetic anisotropy (movie S3) allows dynamic control of the rollers (Fig. 4). In the simplest case, a rotating uniform magnetic field locked on the particle easy axis can be used to control the rolling direction (Fig. 4A). Even in weak external fields of ca. 11 mT, the particles follow the rotating external field, making 90° turns at least down to 200-ms time scales (Fig. 4B). While this expected behavior is dominant, we also observed anomalous responses in a small number of the experiments, where the rotation direction of the particle was opposite to the rotating magnetic field (table S1). However, this happens only in ca. 3% of the cases when all measurements done at different rotation speeds are pooled together (table S1). This suggests the presence of more complex magnetic anisotropies and nontrivial coupling between magnetic electrohydrodynamic forces. Still, the dominant mechanism can be used to program the Quincke rollers to perform nonnatural trajectories, such as square patterns with user-tunable edge widths (Fig. 4C). Even more complex trajectories are possible, either by preprogramming the external field or by using teleoperation using a keypad input device by the experimenter. As a demonstration, trajectories that resemble the letters “SCI” and the Aalto University logo were generated, wherein the combination of ideal external guidance and intrinsic randomness in the Quincke rolling is obvious (Fig. 4D and movie S18).

## DISCUSSION

In conclusion, we have realized magnetic Quincke rollers that allow the rich dynamic behaviors of Quincke rollers to be broadly tuned by using magnetic torques and forces, leading to emergence of active and dimers, anisotropic roller distributions, and collective states in confining potential energy landscapes, programmable rolling patterns, and even teleoperated single-particle dynamics and control. The magnetically created potential energy landscapes offer a third, “ultrasoft,” approach to confining Quincke rollers in addition to the hard liquid-solid walls (*10*) and soft liquid-air interfaces (*12*) reported earlier. The magnetic forces and torques are not used to drive the system into an active state, as done before (*25*, *26*, *39*), but rather to interact with the already active particles (*30*). Hence, these demonstrations suggest that magnetic forces and torques are a powerful and a general tool for controlling and probing the complex dynamics of active systems (*1*, *2*), especially because magnetic nanoparticles can be incorporated relatively easily in both synthetic and biological particles while maintaining their intrinsic motility and interactions. Beyond active matter, we foresee that these demonstrations can also broadly inspire and guide efforts in magnetically tunable electrohydrodynamics (*13*), colloidal self-assembly (*40*), and microrobotics (*41*–*44*). Interesting avenues can be identified also in combining pulsed driving (*11*) to control roller mobility with the magnetic control methods, as well as in using magnetically monodisperse particles with well-defined magnetic anisotropies, such as particles based on single crystalline hematite cubes (*45*, *46*).

## MATERIALS AND METHODS

### Preparation of magnetic Quincke rollers

Magnetic Quincke rollers were created by dispersing commercially available superparamagnetic silica–based microparticles (Microparticles GmbH, SiO2-MAG-AR1054) in n-dodecane (Acros Organics, 99%, anhydrous) containing 150 mM of docusate sodium salt (AOT, Sigma-Aldrich, 99%). Briefly, 100 μl of the aqueous microparticle dispersion was mixed with 10 ml of 2-propanol (IPA, Sigma-Aldrich, 99.8%), followed by collecting the microparticles down at the bottom of the container using a permanent magnet. The supernatant was removed, 10 ml of IPA was added, and the sedimented microparticles were redispersed by shaking. The particles were sedimented for a second time using the same magnet followed by removal of the supernatant. At this point, 10 ml of 150 mM AOT in dodecane was added, and particles were redispersed by shaking and collected down at the bottom of the container once more with the magnet followed by removal of the supernatant. This process using 10 ml of 150 mM AOT in dodecane was repeated two more times. Last, the sedimented particles were redispersed in 2 ml of 150 mM AOT in dodecane. This final dispersion was stored in an open glass vial in a low-humidity chamber (Fisherbrand Desiccator 15594625) at room temperature containing silica gel beads (Sicco V1903-04) that were dried at 100°C for 1 hour in an oven (Memmert). The relative humidity inside the chamber was measured using a logging humidity meter (Testo 174H) to be ca. 5 to 6%. If relative humidity increased above 6%, then the silica beads were replaced with dry ones.

### High-resolution optical imaging of individual magnetic Quincke rollers

High-resolution transmitted light images of individual Quincke rollers were taken with an upright microscope (Zeiss Z1 Imager) equipped with a color complementary metal-oxide semiconductor (CMOS) camera (Ximea MC050CG-SY), a 63×/1.4 oil immersion objective lens (Zeiss) and a 1× tube lens. The sample was prepared by mixing microparticles dispersed in 150 mM AOT in dodecane and microscopy immersion oil (Zeiss Immersol 518 F) in 1:1 ratio and confining the mixture between a regular glass slide (1 mm thick) and a glass coverslip (0.17 mm thick). The oil mixture was used instead of water to better match the refractive index of the particles to the surrounding medium.

### Preparation of planar cells with transparent electrodes for driving the magnetic Quincke rollers

Sample cells consisting of two parallel indium tin oxide (ITO)–coated glass slides (Diamond Coatings, 25 mm by 75 mm, 1.1 mm, 15 to 30 ohms) with a ca. 35-μm gap between the slides were prepared as described earlier (*14*). Briefly, a U-shaped piece of thermoplastic ionomer film (DuPont Surlyn 1702) was placed between the two ITO-coated glass slides. This sandwich structure was placed on a laboratory hot plate (Thermo Scientific HPS RT2 Advanced) with a small weight (ca. 600 g) on top of it. The hot plate was turned on and warmed from room temperature to 130°C. After 10 min at 130°C, the sandwich structure was strongly bonded together and removed from the hot plate. The resulting cell height of ca. 35 μm was confirmed using white light interferometry as described earlier (*14*).

### Magnetic characterization of the magnetic Quincke rollers

The magnetization curve of the magnetic Quincke rollers was measured using a vibrating sample magnetometer (Quantum Design PPMS VSM). The magnetic field applied using the superconducting magnet of the VSM was calibrated by using a palladium reference sample (Quantum Design QDS-C210A) with a mass of 262 mg and a magnetic responsivity of 1.3755 · 10^−2^ emu/T. The sample for magnetometry was prepared by mixing the superparamagnetic silica microparticles dispersed in water with a solution of 5% agarose (Fisher Bioreagents 9012-36-6) in deionized water heated to 50°C in a laboratory oven (Memmert). A small amount of the mixture was collected by capillary action in a rectangular glass capillary (VitroCom 5012-050) where the agarose was allowed to cool down and solidify. The sample was further embedded in ultraviolet (UV) curable adhesive (Norland Optical Adhesive 61) that was solidified in collimated UV light (Thorlabs SOLIS 365C) for 2 min at 1000-mA current. The cured sample was imaged with an optical microscope (fig. S1A) and the number of particles in the sample was manually counted to be 998. The magnetic moment of the sample was measured using the VSM as a function of applied field and divided by the number of particles to estimate the average magnetic properties of a single microparticle (fig. S1B). The magnetization curve was further fitted with the Fröhlich-Kennelly model, yielding the average saturation moment of a single roller to be *m*_s_ = 4.5 ± 0.2 · 10^−8^ emu = 4.5 ± 0.2 · 10^−11^ Am^2^ and the slope near the origin to be *m*/*B* = 2.4 ± 0.1 · 10^−6^ emu/T = 2.4 ± 0.1 · 10^−9^ Am^2^/T.

### Direct evidence of anisotropic magnetic response of individual rollers

A drop of a dilute suspension of the superparamagnetic particles (dispersed in water) was confined in a horizontal Hele-Shaw cell that was placed in a uniform and horizontal rotating magnetic field generated by two pairs of Helmholtz coils with orthogonal axes (Serviciencia Ferronato BH300-3-G). The coil pairs were driven by two independent power supplies (Kepco BOP 72-6 M) controlled using LabView to generate a rotating field $B(t)=B_{0}[\sin(2\pi ft)\hat{i}+\cos(2\pi ft)\hat{j}]$, where *B*_0_ = 2 mT and *f* = 250 mHz. The sample was observed using a custom optical imaging system constructed of standard optomechanical components (Thorlabs), an infinity-corrected objective lens (Nikon LU Plan 100×/0.80), a tube lens (Nikon CM-20 0.5×), and a CMOS camera (Point Grey Grasshopper GS3-U3-51S5M-C). Individual particles were observed to rotate with the rotating magnetic field, suggesting the existence of a preferred direction of magnetization, i.e., anisotropy.

### Experiments in static uniform magnetic fields

Static uniform magnetic fields were created using a pair of electromagnet coils and a power supply as described earlier (*47*). Briefly, the two coils (GMW Associates 11801523 and 11801524) were positioned vertically with a gap of 50 mm between them using standard optomechanical components (Thorlabs). The coils were excited using a laboratory power supply (BK Precision 9205). Imaging was done using a homemade microscopy setup consisting of standard optomechanical components (Thorlabs), an infinity-corrected objective lens (Nikon Plan Apo 2×), a tube lens (Thorlabs TTL100-A), and a CMOS camera (Point Grey Grasshopper GS3-U3-51S5M-C). High-speed and high-magnification imaging was done using a homemade microscopy setup consisting of standard optomechanical components (Thorlabs), an infinity-corrected objective lens (Nikon LU PLAN 100×/0.80), a tube lens (Nikon CM-20 0.5×), and a high-speed camera (Phantom Miro M310).

### Experiments in spatially varying magnetic fields

Various permanent NdFeB magnets were used to create the magnetic energy landscapes. A large cylindrical magnet with a diameter of 25.4 mm and height of 25.4 mm (K&J Magnetics DX0X0-N52) was used to create the approximately uniform magnetic field gradients. A cylindrical NdFeB magnet with a diameter of 6.4 mm and height of 12.7 mm (K&J Magnetics D48) and smaller ring-shaped magnets (K&J Magnetics R422CS and R311) were used to create the axisymmetric potentials. A slab-shaped rectangular magnet magnetized through its middle breadth (K&J Magnetics BC14-N52) was used to create the linear magnetic trap. The magnetic fields generated by the magnets were measured using a three-axis gaussmeter (SENIS AG 3MTS). The distance of each magnet from the Quincke rollers was controlled using a motorized stage (Thorlabs) and spacers of known thickness. Imaging was done using optical microscopes (Leica Z16 APO and Nikon CM-20 0.5×) in transmitted and reflected modes. The orientation of the particles’ motion was determined using particle image velocimetry [PIVlab for MATLAB; (*48*)].

### Experiments in dynamic uniform magnetic fields

Dynamic uniform magnetic fields for producing time-variable magnetic torques were generated with two cylindrical NdFeB permanent magnets (K&J Magnetics DX0X0-N52) mounted on a dovetail rail (Thorlabs XT66SD-250) at a distance of 132 mm from each other, producing an approximately uniform field of 11.2 mT at the midpoint between the magnets measured using a gaussmeter (SENIS AG). The dovetail was attached to a planetary geared DCmotor (Micro Motors SRL E192.24.25) that was driven with a motor driver (Seeed Studio MD13S) that was programmed using a microcontroller (Arduino Uno) connected to a PC, allowing motor control using custom scripts and a membrane keypad. The motor and the driver were powered using a benchtop DCpower supply (Multicomp Pro MP710086) set at 24 V and 5 A. Pulse-width modulation (PWM) was used to control the rotation speed of the dovetail and, hence, the direction of the magnetic field. The rotation speed as a function of PWM was determined by direct imaging using a compact camera (Nikon J1). The motor system decoupled from the imaging system by constructing these systems on adjacent tables. The sample cell was held stationary in the midpoint between the rotating magnets using optomechanical components (Thorlabs). Imaging was done with a homebuilt microscope consisting of an infinity-corrected objective lens (Mitutoyo 5×/0.14), a tube lens (Thorlabs), a CMOS camera (Basler acA2440-75um) and coaxial illumination using white LED (Thorlabs MCWHLP1) using a beam splitter (Thorlabs BSW10R).
