# Supplementary material for: Magnetic Quincke rollers with tunable single-particle dynamics and collective states
Source: Sci Adv. 2023 Jun 30;9(26):eadh2522. doi: 10.1126/sciadv.adh2522 (PMC10313172; doi:10.1126/sciadv.adh2522)
Supplement: Supplementary file 1 — Figs. S1 to S4 Table S1 Legends for movies S1 to S18 [file sciadv.adh2522_sm.pdf]

Science Advances
AAAS

Supplementary Materials for

Magnetic Quincke rollers with tunable single-particle dynamics and collective states

Ricardo Reyes Garza *et al.*

Corresponding author: Jaakko V. I. Timonen, jaakko.timonen@aalto.fi

Sci. Adv. **9**, eadh2522 (2023)
DOI: 10.1126/sciadv.adh2522

The PDF file includes:

Figs. S1 to S4
Table S1
Legends for movies S1 to S18

Other Supplementary Material for this manuscript includes the following:

Movies S1 to S18

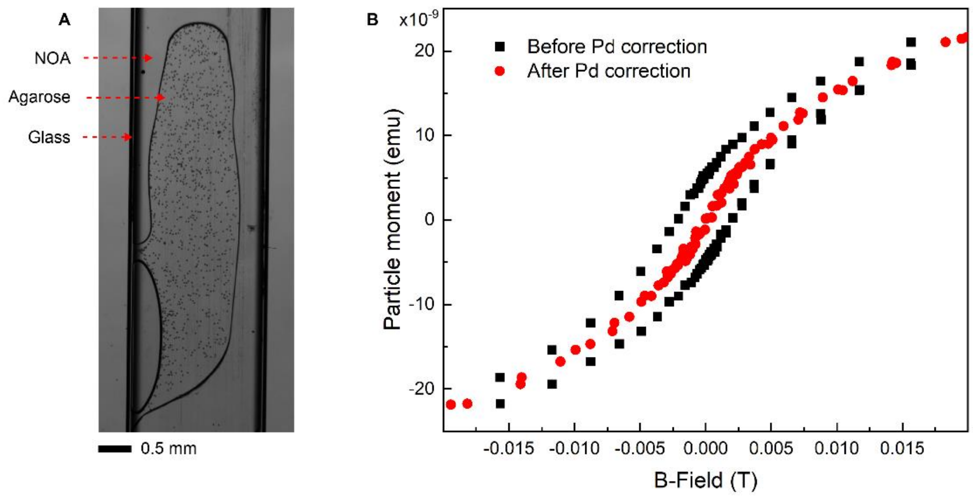

Fig. S1.

Magnetic characterization of the magnetic Quincke rollers. (**A**) A microscopy image of the magnetometry sample containing N = 998 magnetic silica microparticles embedded in agarose gel inside a rectangular glass capillary tube. (**B**) A plot showing the average magnetic moment per particle as a function of the applied magnetic field before and after the correction of the B-field with the Pd calibration.

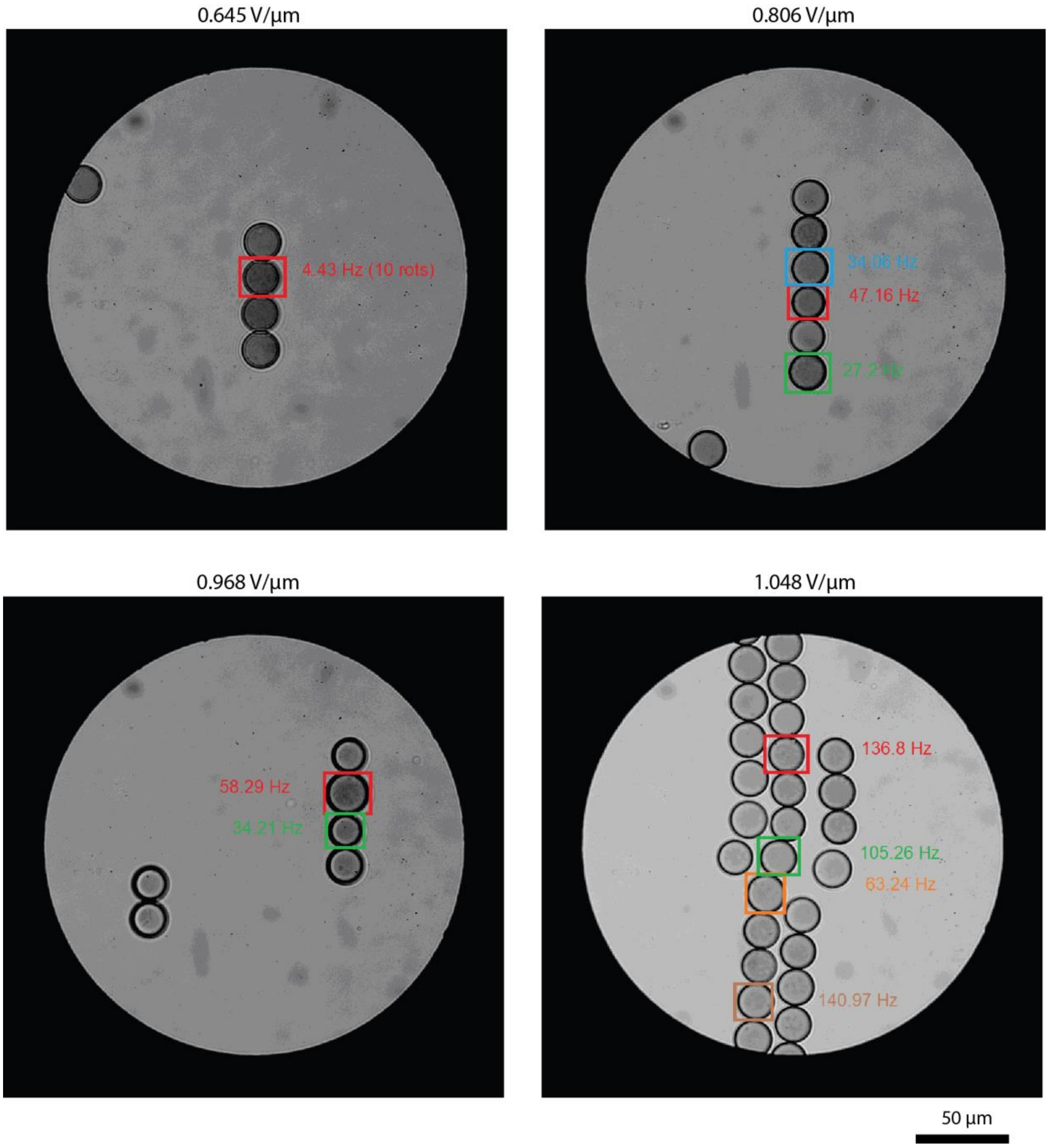

Fig. S2.

Rotation frequencies of magnetic Quincke roller chains. Measured rotation frequencies for randomly chosen particles for four different values of the electric field, done with high-speed camera imaging. The cell height was h= 30.9±1.6 μm. There is an increase in rotational speed with increasing electric field magnitude (as expected by Quincke rotation), but not all the particles in a chain rotate at the same speed. Additionally, the frequencies measured indicate a large amount of slip, with the particles almost completely hovering mid-fluid.

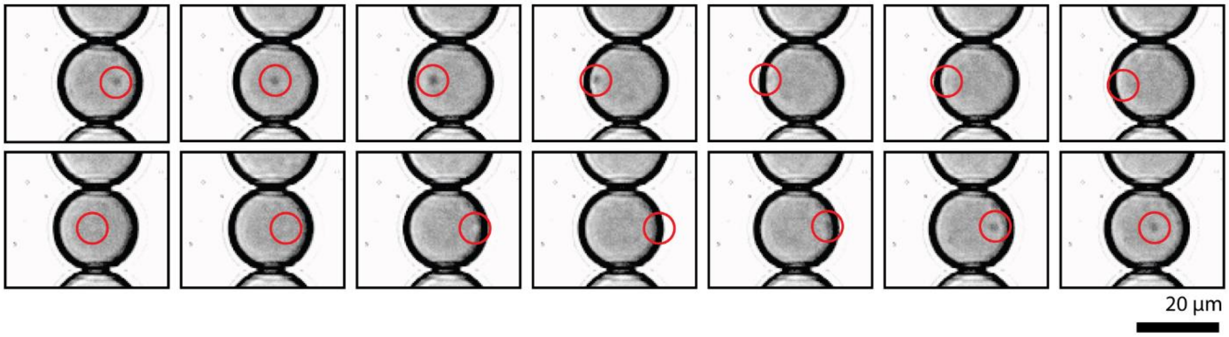

Fig. S3.

Determination of rotation axis and frequency. An image sequence showcasing the motion of a typical defect on the surface of a single magnetic Quincke roller in a chain as a function of time (E_0= 0.81 V/µm). The defect, in the center of the red circle, appears dark when moving from right to left (upper row) and subsequently turns light gray as the motion continues from left to right (lower row). The change from dark to light (and back to dark) indicates change of the position of the defect along the imaging axis. The data was collected at 3200 fps and every 5th frame is shown, indicating that the time difference between neighboring frames is 1.56 ms and the full rotation takes place in ca. 20 ms (corresponding to frequency of ca. 50 Hz). The axis of rotation is perpendicular to the horizontal line travelled by the defect.

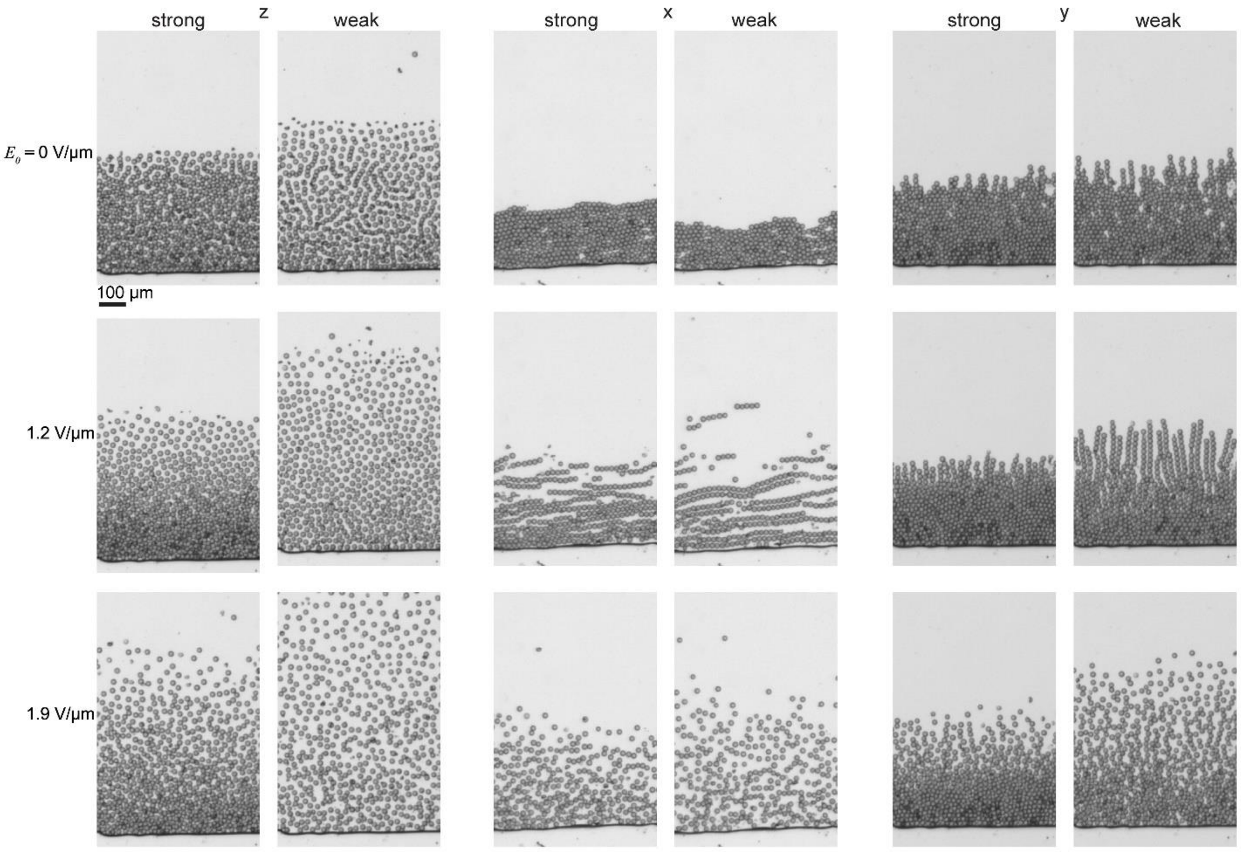

Fig. S4.

Effect of the magnetic field strength and gradient on the collective states in confining potentials. Images showing snapshots of the steady-states of the rollers in strong and weak confining potentials at different driving fields and magnetic field directions (x, y, z). The magnetic field strength and magnetic force in the "strong" cases are the same as the ones shown in **Fig. 3(A-C)**. For the weak z and x cases, the field decreased from 57 mT to 49 mT over a distance of 1200 μm, corresponding to a gradient of 7 mT/mm, producing a magnetization of the particles between 75% and 71% of saturation, and leading to an approximately constant force $F_B \sim 0.3$ nN. For the weak y case, the field decreased from 158 mT to 135 mT through the same distance, 1200 μm, as in the previous cases, corresponding to a gradient of 19 mT/mm, producing a particle magnetization between 89% and 87% of the saturation, and a force $F_B \sim 0.8$ nN. Particles closer to the magnet tend to pack tighter when the magnetic force is stronger, while particles further away tend to move more freely when the magnetic force is reduced.

PWM (%)	t_{90} (ms)	ω (deg/s)	N_{CW}	N_{CCW}	N_{other}
100	150	600	18	2	3
88.2	170	529	12	0	0
72.5	205	439	15	0	2
54.9	225	400	15	0	3
39.2	325	277	10	1	2
23.5	460	196	14	0	2
17.6	645	140	8	0	0
11.8	1020	88	11	0	4

Table S1.

Summary of right-angle turning of Quincke rollers in rotating magnetic field. Table shows the motor power setting controlled using pulse width modulation (PWM), time required for the motor to make a 90 degree rotation clockwise (t_{90}), the corresponding mean angular velocity (ω), the number of observed clockwise rotations of the Quincke rollers (N_{CW}), the number of observed counter clockwise rotations of the rollers (N_{CCW}), and the number of other observed particle behaviors including the particle being stuck, spinning or not responding to the magnetic field (N_{other}). Out of all observed rotations ($N_{\mathrm{CW}} + N_{\mathrm{CCW}} = 106$) only three correspond to the anomalous case ($N_{\mathrm{CCW}} = 3$), i.e. ca. 3%.

Movie S1.
Normal rolling of magnetic Quincke rollers in absence of magnetic field (**Fig. 1C**).

Movie S2.
Back-and-forth rolling (twitching) of the magnetic Quincke rollers in absence of magnetic field (**Fig. 1C**).

Movie S3.
Particle rotating in a rotating magnetic field in the absence of electric field (anisotropic magnetic response).

Movie S4.
Assembly of magnetic Quincke rollers into chains upon application of a uniform magnetic field for different electric field values, and disassembly when magnetic field is turned off (**Fig. 2A**).

Movie S5.
Normal active chains, high speed imaging (**Fig. 2B**).

Movie S6.
Anomalous roller dimer, high speed imaging (**Fig. 2B**).

Movie S7.
Anomalous roller dimer, translational motion (**Fig. 2B**).

Movie S8.
Active chain fusion (**Fig. 2D**), fragment transfer (**Fig. 2E**), and scattering (**Fig. 2F**).

Movie S9.
Rollers in uniform magnetic field gradient 1, z direction (**Fig. 3A**).

Movie S10.
Rollers in uniform magnetic field gradient 2, x direction (**Fig. 3B**).

Movie S11.
Rollers in uniform magnetic field gradient 3, y direction (**Fig. 3C**).

Movie S12.
Diluted system of rollers in uniform magnetic field gradient 1, z direction.

Movie S13.
Diluted system of rollers in uniform magnetic field gradient 2, x direction.

Movie S14.
Diluted system of rollers in uniform magnetic field gradient 3, y direction.

Movie S15.
Rollers in linear trench-like and circular racetrack-like potentials (**Fig. 3D**).

Movie S16.
Rotating roller condensate in axisymmetric potential (**Fig. 3F**, **G**).

Movie S17.
Alternating direction of rotation of the roller condensate (**Fig. 3H**, **I**).

Movie S18.
Teleoperated control of a single roller (**Fig. 4D**).
